# Advances in the biology of cerebral cavernous malformations

**DOI:** 10.4103/2152-7806.70962

**Published:** 2010-10-11

**Authors:** Jason S. Hauptman, Parham Moftakhar, Andrew Dadour, Dennis Malkasian, Neil A. Martin

**Affiliations:** Department of Neurosurgery, David Geffen School of Medicine at the University of California, Los Angeles, USA; 1 Department of Radiology and Biomedical Imaging, University of California, San Francisco, CA, USA

**Keywords:** Angioarchitecture, cavernous malformation, CCM, immunology, molecular biology

## Abstract

**Object:**

To provide a review of current, high-impact scientific findings pertaining to the biology of cerebral cavernous malformations (CCMs).

**Methods:**

A comprehensive literature review was conducted using PubMed to examine the current literature regarding the molecular biology and pathophysiology of CCMs.

**Results:**

In this literature review, a comprehensive approach is taken to review the current scientific status of CCMs. This includes discussion of molecular biology and animal models, ultrastructure and angioarchitectural features and immunological methods and hypotheses.

**Conclusions:**

Studies examining the molecular biology of CCMs have shown that genes involved in angiogenesis, blood–brain barrier formation, cell size regulation, vascular permeability and apoptosis play critical roles in the ontogeny of this disease. *In vivo* work suggests the likelihood of a “two-hit mechanism” resulting in somatic mosaicism and biallelic loss of angiogenic genes. The etiological effects of angioarchitecture and immune response within these lesions further complicate the pathophysiology. Future treatment endeavors will necessitate exploitation of the multiple facets of CCM formation to maximize success at CCM prevention or obliteration.

In our following review, we attempt to identify key advances in the understanding of the pathogenesis and behavior of cerebral cavernous malformations (CCMs). There is no doubt that the spectrum of vascular pathologies incur a tremendous burden on neurosurgical healthcare delivery, with many vascular insults changing our patients’ lives in ways that impact their ability to conduct daily routine activities. Cerebral cavernous malformations, as an example, have a relatively high prevalence (as far as vascular malformations are considered) and create functional problems for our patients such as motor deficits, sensory problems, and epilepsy. As surgical neuroscience continues to expand to into the domain of molecular medicine and genetic technology, it is more important than ever to gain a deeper understanding of these malformations in the hopes to provide more efficient methods of detection and treatment.

In the paper, we discuss several genes implicated in the development of CCMs. These genes play critical roles in the development and function of the normal cerebrovascular system, and disruption of these genes’ functions play critical roles in the etiology of these vascular malformations. Additionally, work describing the ultrastructure of CCMs suggests that their development is not only impacted by dysfunctional genes, but also by the presence of developmental venous anomalies and altered hemodynamics within and around these lesions. The role of immune response in the etiology of CCMs is only beginning to be explored, with mounting evidence suggesting the body’s humoral response to these lesions may be contributing to CCM proliferation and/or hemorrhage.

As neurosurgeons, we are constantly adapting methods of diagnosis and treatment of our patients to keep up with evolving technology and scientific progress. The basic science underlying CCM and other vascular malformation development should be no different. While it may appear at first glance that rodent knockout models and cellular biology are a far cry from the craniotomy for CCM resection, they are not as distant as one might think. Examining the genomes and resected lesions from CCM families resulted in the discovery of the CCM genes. Genetic screens are already available to identify individuals at risk for familial CCMs. As our molecular understanding of these lesions progresses, so will our ability to detect these lesions earlier, before they are symptomatic. Furthermore, the cellular biology of the altered blood vessel growth and proliferation in CCMs will identify potential targets for drugs that may inhibit the proliferation of these lesions. The role of the immune response is particularly exciting, and raises the possibility of immunomodulating therapy in the treatment regimen of CCMs.

Our neuroscience and neurosurgical colleagues in the field of vascular malformations are conducting terrific and exciting work. The science potentiates our enthusiasm to be training in the age of molecular medicine, and as such we eagerly anticipate further application in the clinical arena. Our review of their work is not only homage to their diligent and industrious labors, but also an eager attempt to keep busy neurosurgeons “in the loop,” particularly as the science progresses at an exponential pace.

## INTRODUCTION

Vascular malformations incur a substantial economic burden on the healthcare system.[34] Cerebral cavernous malformations (CCMs) are a relatively frequent pathology encountered in vascular neurosurgery, with an incidence of approximately 1 in 200.[37] CCMs consist of dilated capillaries composed of a single layer of endothelial cells with high vascular permeability, lending them susceptible to repeated rupture and adjacent neuronal injury. While various techniques exist to help clinicians identify and treat these lesions, their etiology and pathophysiology are only recently beginning to become elucidated.

Understanding the molecular biology of CCMs is critical to developing novel diagnostic modalities (targeting the underlying mutation, such as genetic screens for heritable cavernomatosis) and therapies. In this review, we present an update as to the prevailing scientific endeavors aimed at understanding how CCMs are formed and how to best diagnose them. In the first section, current literature regarding genetic dysfunction in these lesions is reviewed. Next, relevant studies examining the ultrastructure and angioarchitecture of CCMs are discussed. Finally, hypotheses and experiments demonstrating the role of the immune response in the pathology of CCMs are presented.

## MOLECULAR BIOLOGY OF CCMS AND THE TWO-HIT HYPOTHESIS

Understanding the molecular biology of CCMs is critical to revealing the etiology of these pathological entities as well as developing targeted genetic approaches for therapy. Most of the initial work in discovering genes implicated in this disease has come from screening of families with a known heritable CCM phenotype. This has led to the development of molecular screening tests that have been used successfully to identify genetic anomalies in cases of familial CCMs.[38] These screens can be particularly useful in guiding management of CCMs as some authors suggest surveillance of the brain and spinal cord in families with known heritable cavernous malformation syndromes.[28]

The three genes that have been the focus of a majority of CCM research are Krit1/CCM1, MGC4607/CCM2 and PDCD10/CCM3 [Table 1]. Together, mutations in these genes account for over 85% of familial CCM syndromes, with Krit1/CCM1 and MGC4607/CCM2 accounting for the majority (each ~40%).[30] The current thought is that the CCM genes play a role in angiogenesis and vasculogenesis.[56] All three genes are expressed throughout the neuronal cell layers during development and adulthood as well as within the developing blood vessels by mid-gestation.[41] MGC4607/CCM2 and PDCD10/CCM3 in particular are robustly expressed within the meningeal and parenchymal cortical vessels post-natally.

**Table 1:** Genes implicated in the pathogenesis of CCMs

Gene	Location	Putative functions
Krit1/CCM1	7q21	Angiogenesis (integrin-dependent), blood–brain barrier formation, cell–cell adhesion, cell size and shape (via interactions with cytoskeletal elements)
MGC4607/CCM2	7p13-15	Angiogenesis, blood–brain barrier formation, Rho-dependent vascular permeability
PDCD10/CCM3	3q26-27	Unclear, induces apoptosis within endothelial cell lines
Pten	10q23	Regulates cell size, cell growth and proliferation, dendritic arborization, cell motility

**Figure 1:**
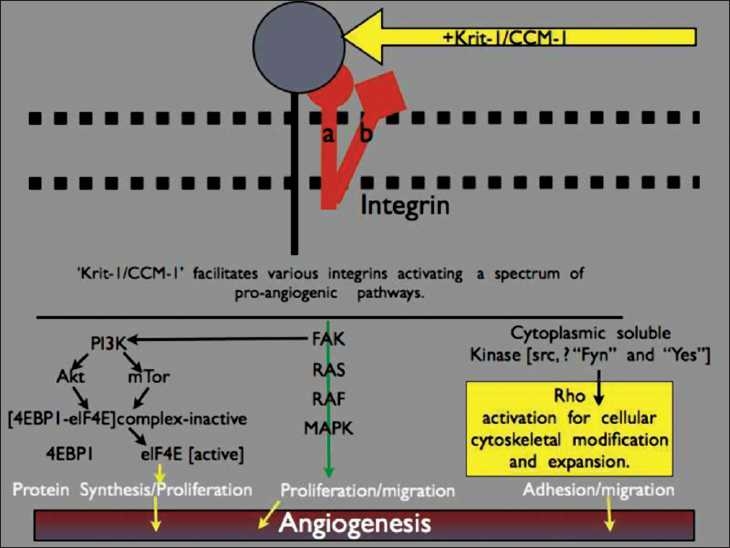
Schematic of the role of Krit-1/CCM1 in angiogenesis. Krit-1/CCM-1 interacts with integrins that in turn facilitate the pro-angiogenic cascade. This includes activation of pathways involved in protein synthesis, proliferation, and adhesion

Krit1 (Krev-1 interaction trapped 1)/CCM1 is located on chromosome 7q21.[10,20,49] Krit1/CCM1 was initially identified as a putative tumor-suppressor gene, endogenously expressed at low levels and acting to inhibit Ras activation through an interaction with Krev-1 (Kirsten-ras-revertant 1).[27,49] Krev-1 is though to antagonize cell growth in response to G-protein activation from negative growth-regulatory signals.[26] Krit1/CCM1 encodes a microtubule-associated protein that likely directs cytoskeletal structure and helps to determine endothelial cell size, shape and function.[19] Krit1 also plays a role in cell–cell adhesion, possibly explaining the enhanced permeability of CCMs and propensity for hemorrhage.[62] This is likely integrin dependent, with work demonstrating Krit1/CCM1’s function in beta1 integrin-dependent angiogenesis [Figure 1].[35,63] Immunostaining for Krit1/CCM1 in multiple organs has revealed positivity in the endothelium of capillaries and arterioles, particularly in areas where a blood-organ barrier exists.[20] In the brain, expression of Krit1/CCM1 is found within astrocytic foot processes and cortical pyramidal neurons in addition to the vascular endothelium, suggesting a role in angiogenesis and blood-brain barrier formation.

MGC4607/CCM2 loss accounts for up to 38% of familial CCM cases.[23,30] This gene is located on chromosome 7p13-15.[13] MGC4607/CCM2 has a similar temporal and spatial expression pattern as Krit1/CCM1 within the endothelium of the arteries.[48] Just like Krit1/CCM1, MGC4607/CCM2 is also expressed in astrocytic foot process and pyramidal neurons, suggesting a putative role in blood-brain barrier formation. It is thought that MGC4607/CCM2 regulates RhoA activity, in turn affecting vascular permeability. One way this occurs is by appropriately localizing a ubiquitin ligase called Smurf1, which is responsible for the normal degradation of RhoA, and thus maintain normal vascular endothelial cell function.[14] Animal models have demonstrated that constitutive MGC4607/CCM2 loss results in fetal non-viability, while MGC4607/CCM2 deletion in neuroglial precursor cells has no observable neural phenotype. When it is specifically knocked out of the vascular endothelial cells, however, angiogenesis is disrupted and vascular anomalies can be detected.[6]

Importantly, the physical interaction between Krit1/CCM1 and MGC4607/CCM2 appears to be essential for proper formation of endothelial cell–cell junctions and regulation of vascular permeability.[53] This has important therapeutic implications as the application of fausidil, an inhibitor of the downstream RhoA/ROCK pathway (which is otherwise constitutively activated when either MGC4607/CCM2 or Krit1 is inactivated), reverses the increase in vascular permeability noted in Krit1/CCM1 and MGC4607/CCM2 heterozygous mutants. Inhibitors of this activated pathway may thus restore endothelial cell–cell junctions and reduce hemorrhage in familial cavernous malformation syndromes where the RhoA/ROCK pathway is upregulated.

Another mutation implicated in the pathogenesis of familial CCM disorders is PDCD10/CCM3, although this is found least commonly in screens of CCM families.[5,18] This gene has been localized to chromosome 3q26-27, having been identified by screening multiple families with a heritable CCM phenotype. PDCD10 (programmed cell death gene 10) displays an embryonic expression pattern similar to both Krit1/CCM1 and MGC4607/CCM2, particularly localizing to the vascular endothelium and neurovascular unit.[55] Notably, it is regulated by the oncogenic transcription factor c-Myc,[12] which could be another avenue by which PDCD10/CCM3 functional activity is altered. Specific activities of the PDCD10/CCM3 gene are still currently being explored. Although *in vitro* overexpression of PDCD10/CCM3 induces apoptosis, inhibition of the gene decreases cell death by activating caspase-3.[11] Chen *et al*. have hypothesized that mutations affecting PDCD10/CCM3 result in anomalous apoptosis (or lack thereof) within the neurovascular unit thus altering the ultrastructure of the underlying capillaries.

Another previously unrecognized gene that appears to play a role in CCM formation is Pten (phosphatase and tensin homolog on chromosome 10).[64] Pten is known to play a crucial role in the regulation of cell growth and proliferation as well as programmed cell death.[17,29,33] Work by Zhu *et al*. demonstrates that Pten loss may occur in up to one-third of the endothelial cells within the CCMs, particularly in cases where the lesions are multiple and/or small. Interestingly, while many cell growth and soma size regulatory activities guided by Pten are regulated by upstream activation of PI3K, Pten activity within CCMs seems to be independent of this pathway. While it is known that Pten plays a role in angiogenesis and endothelial cell proliferation,[22] its role in the etiology of anomalous vascular lesions is just beginning to be elucidated.

**Figure 2:**
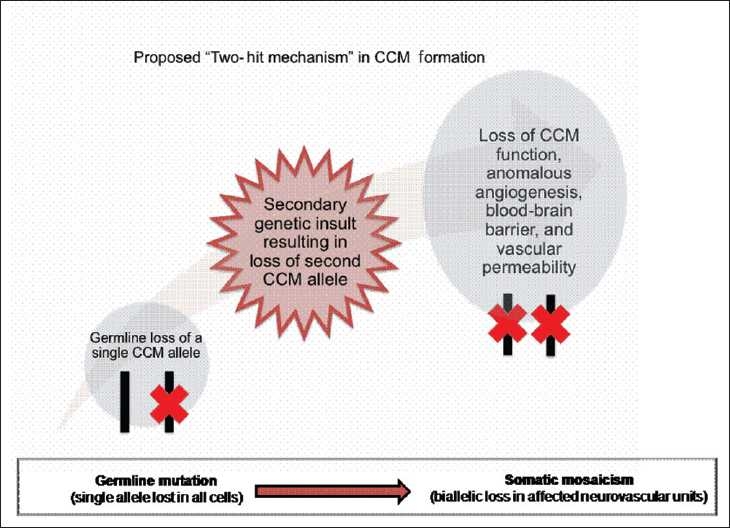
Schematic of the proposed two-hit hypothesis underlying generation of CCMs

Work in resected CCMs from patients with known familial disease (i.e., germline mutations in one of the three CCM genes) has demonstrated that development of CCMs requires biallelic loss of the affected gene within the affected endothelial cells but not in the surrounding normal or reactive endothelial cells.[37] This is further supported by work by Akers *et al*. showing the presence of both germline and somatic loss of the CCM genes in resected lesions.[3] Both authors clearly demonstrate that CCM formation will result from complete inactivation of one of the CCM genes. This work also suggests that mosaicism of loss of function mutations within endothelial cells is the likely cause of CCM formation and also raises the possibility of a two-hit mechanism for mutations [Figure 2].

This notion of the “two-hit hypothesis” has been demonstrated in mouse models, where mice heterozygous for Krit1/CCM1 loss and homozygous for p53 loss (thus increasing the rate of somatic mutation of Krit1/CCM1) developed CCMs.[42] Although some mice heterozygous for MGC4607/CCM2 loss develop CCMs, the penetrance is low, consistent with the hypothesis that somatic mosaicism may be necessary in order for these lesions to form.[43] Other lines of evidence supporting the notion of the two-hit mechanism include the development of *de novo* CCMs in children several years after receiving cranial radiotherapy for unrelated malignant disease.[54] A recent report from Motegi *et al*. has also demonstrated *de novo* formation of a CCM in a patient 80 months after receiving radiosurgery for a basal ganglia arteriovenous malformation (AVM).[36] This suggests than an additional insult to local endothelial cells may result in the “second hit” and loss of angiogenic tumor suppressor genes (i.e., CCM genes).

## ULTRASTRUCTURE FEATURES AND ANGIOARCHITECTURE OF CCMS

CCMs are classically described as discrete well-circumscribed lesions comprising sinusoidal spaces lined by a single layer of endothelium. A collagenous matrix devoid of elastin, smooth muscle or other vascular wall elements separates them. Traditionally, they were considered to be “static” vascular lesions, as opposed to arterial vascular malformations (AVMs), which often have complex flow patterns. However, recent investigations in the angioarchitecture of CCMs suggest that these vascular lesions can no longer be considered as just “static.”

Much of what has recently been uncovered about the angioarchitecture of CCMs comes from its association with developmental venous anomalies (DVAs). Coexistence of DVAs with CCMs is common, with the association reported in 24–86% of CCMs.[1,44,45,61,65] Several of the reported cases have been of de novo CCMs in the drainage territory of the DVAs.[4,8,9,31,60] There is a 12.5–28.3% incidence of white matter signal abnormalities proximal to a DVA on magnetic resonance imaging, which is thought to present early stages of CCM development.[46,47] It is thought that the abnormal vascular anatomy of the DVAs may lead to venous hypertension, which in turn leads to formation of CCMs due to angiogenic proliferation. This is often referred to as the “hemorrhagic angiogenic proliferation” hypothesis.[57]

A recent study by Hong *et al*.[21] went even further and demonstrated how specific angioarchitectural features of DVAs could result in flow disturbances leading to a cascade of events leading up to the development of CCMs. In every patient with concomitant CCMs and DVAs examined in their study, Hong and colleagues noticed that there was acute angulation of the distal medullary or draining vein. The angulated course of the blood vessel could bring on steep changes in the direction or speed of blood flow, leading to vessel wall injury by turbulent flow and making it prone to hemorrhage. A second factor that the authors noted was narrowing of the diameter of the distal draining vein. Narrowing of the pathway of blood flow would result in hemostasis and increasing venous pressure. The third factor, severe tortuosity, of the draining vein could also result in blood flow stasis. All the above factors resulting in elevated venous pressures could overload the vessel wall leading to microhemorrhages from the weakest point, in turn leading to reactive angiogenesis.

Cavernous malformations associated with visible DVAs may have an increased incidence of hemorrhage and non-hemorrhagic neurological symptoms as compared with CCMs without visible DVAs. The incidence rate of hemorrhage ranges from 62% to 93% in patients with CM with an associated DVA[1,58,61] as compared with 38% in patients with CCMs without visible DVAs[60] or 0–0.68% in patients with DVA alone.[15,16,32,39] When the CCMs and DVAs coexist, the CCMs are likely sporadic as opposed to familial. In fact, no visible DVAs adjacent to CCMs have been reported in patients with familial CCMs, suggesting a different possible pathogenesis between familial and sporadic forms.[2,40]

## ROLE OF THE IMMUNE RESPONSE IN CCMS

A potential role of the immune system in CCMs has just recently been revisited. Macrophage inflammatory cells have long been recognized to infiltrate CCM lesions, especially in reaction to acute bleeding.[59] However, the involvement of the humoral system in CCMs has recently been investigated. The CCM phenotype predisposes to vascular leakage and accumulation of blood products in the adjacent brain tissue. This phenomenon may create a special milieu for antigenic challenge and immune response.

Shi and colleagues,[51] through isoelectric focusing studies, demonstrated that CM lesions had oligoclonal patterns of IgG unrelated to peripheral blood contamination, indicating selective synthesis of IgG within these lesions. Additionally, the demonstration of IgG of restricted heterogeneity within CM lesions suggests that the immune response is produced by a limited number of plasma cell clones. Normally, immunoglobulins (Igs) are not synthesized within the central nervous system (CNS), but when B cells enter the CNS – if they proliferate in response to local antigen stimulation – they may undergo clonal expansion and differentiate into plasma cells.

Oligoclonal IgG bands can be identified in the CSF of patients with a variety of infectious and inflammatory conditions involving the nervous system. The repeated hemorrhages in CCMs could also lead to a chronic inflammatory state and, like other CNS disorders, stimulate a humoral response. That being said, neither lesion growth nor hemorrhage appears to be necessary for immune cell infiltration, raising the possibility that the endothelium of the CCMs itself is antigenic.[52] The nature of the immune reaction also seems to differ based on the architecture of the CCM – those CCMs associated with venous anomalies seem to incur a more robust humoral response (i.e., B-cell infiltration) as opposed to solitary lesions that tend to instigate T-cell expansion.[52]

The work by Shi and colleagues also demonstrated the absence of oligoclonal IgG in AVMs.[51] The AVMs lack the leaky endothelial cell layer and repetitive hemorrhages associated with CCMs, or the cluster of organized clot in brain parenchyma. Such features of CCMs may reflect the characteristic immune response apparently associated with CCMs but not with AVMs.

An intralesional humoral response in CM lesions could explain, in part, why some CCMs remain biologically dormant, whereas others proliferate with serious clinical consequences. This data can stimulate future projects to determine the sequences for the Ig light chains. Artificial constructs for antibodies, derived from a monoclonal sequence, would be used to localize and possibly identify antigenic triggers involved in CCMs. Given the characteristic vascular phenotype associated with CCMs – thrombus of varying ages sequestered within “caverns,” a leaky blood–brain barrier and chronic deposition of iron and blood degradation products, it would not be surprising that an immune response could play a contributory role to the pathogenesis of CCMs.[50]

A causal relationship between immune response and lesion growth of CCMs cannot be proven by the present data. However, an immune response has been associated with other processes of vascular proliferation, including rheumatoid arthritis, inflammatory bowel disease and multiple sclerosis.[7,24,25] These other pathologies could serve as a model for future studies of immunology in CCMs. Clearly, more work needs to be undertaken in existing CCM models, such as the murine CCM knockouts.

## CONCLUSIONS

While significant work has been performed to characterize the biologic nature of CCMs, many questions are left unanswered. Emerging transgenic murine models of CCMs involving spatial and temporal deletion of the CCM genes will assist in further understanding of the roles that Krit1/CCM1, MGC4607/CCM2 and PDCD10/CCM3 play in normal angiogenesis/vasculogenesis as well as pathological formation of vascular lesions. Clearly, other tumor suppressor and/or protooncogenes, such as Pten, must be involved in CCM formation as well because ~15% of the familial forms do not have CCM gene deletion (in addition to sporadic forms of CCMs). The angioarchitecture of these lesions also complicates their natural evolution, although the activation of cascades involve vascular growth factors and hypoxia-induced pathways. Further, adding to the complexity of the in vivo behavior of CCMs is the immune response to either the anomalous endothelial cells themselves or the resultant hemorrhage, leading to a chronic inflammatory state within or adjacent to these lesions. Future treatment modalities will need to exploit all of the features of CCM pathophysiology in order to successfully remedy or prevent these lesions from forming.

It is reasonable to hypothesize that knowledge concerning the biology of CCMs will lead to developments in diagnosis and treatment of these lesions. High-throughput molecular screens and genetic testing will aid in the identification of chromosomal aberrations leading to both familial and sporadic forms of CCMs. Early identification of genetic abnormalities in families will also lead to enhanced screening for undiagnosed vascular lesions before they hemorrhage. This will, of course, enable patients to have their lesions removed before they hemorrhage and causes deficit and/or epilepsy. Exploring the pathways of aberrant genes within cavernous malformations may also lead to treatments with pharmacological agents that modulate the Krit1/CCM1, MGC4607/CCM2 and PDCD10/CCM3 pathways, resulting in potential stasis or involution of CCM lesions. An example of this is fausidil, which may play a role in the treatment of CCM lesions in which the RhoA/ROCK pathway is upregulated. Beyond the molecular biology of these lesions, the role of the immune system may also have an interesting impact on the treatment of CCMs. If future work demonstrates a clear relationship between the immune response and the growth of CCMs, there may be a role of immunosuppressant therapies in managing patients with multiple CCMs that are being followed over time. In all, this is an exciting time in vascular biology as we begin to understand the nuances of vasculogenesis and angiogenesis and harness this knowledge to develop adjunctive modalities for diagnosis and treatment of vascular lesions.
